# Clinical features of failure of conservative management in adult patients with adhesive small bowel obstruction: a retrospective cohort study

**DOI:** 10.3389/fmed.2026.1870352

**Published:** 2026-07-20

**Authors:** Jie Shen, Bin Chen, Hongyang Xie, Yiping Wang

**Affiliations:** Department of Gastrointestinal Surgery, Ningbo Municipal Hospital of Traditional Chinese Medicine (TCM), Affiliated Hospital of Zhejiang Chinese Medical University, Ningbo, China

**Keywords:** conservative management, emergency, laparotomy, risk factor, small bowel obstruction

## Abstract

**Background:**

Adhesive small bowel obstruction (ASBO) is a common cause of acute abdominal pain in adults. Most patients with ASBO without strangulation are successfully managed conservatively. However, some patients fail nonoperative treatment, develop intestinal ischemia and undergo surgical management. The clinical features of these patients require further investigation.

**Methods:**

Patients who received conservative management as the initial treatment for ASBO between January 2017 and December 2025 were selected from the electronic medical records of a single institution. The patients were categorized into a successful conservative treatment group (group A) and a surgery group (group B). Demographic and clinical variables were compared between the two groups.

**Results:**

A total of 510 patients were included in this retrospective study. Groups A and B comprised 456 and 54 patients, respectively. Univariable analysis demonstrated a significant difference in five factors: age, multiple previous operations, C-reactive protein (CRP) level, body temperature, and maximum diameter of the dilated small bowel (MDSB) (*P* < 0.05). Multivariable logistic regression analysis indicated that older age (OR 1.035, *P* = 0.019), higher CRP level (OR 1.024, *P* = 0.003), elevated body temperature (OR 9.812, *P* < 0.001), and larger MDSB (OR 4.860, *P* < 0.001) were independent risk factors for failure of conservative management of ASBO. Receiver operating characteristic curves were plotted to evaluate the predictive age, CRP level, body temperature, and MDSB values, yielding areas under the curve of 0.6122, 0.6337, 0.8193, and 0.7052, respectively. Patients in group B had longer hospital stays, higher hospitalization costs, and more intensive care unit admissions than those in group A. However, no statistical difference was observed in the post-discharge recurrence rate.

**Conclusion:**

Our study established older age, higher CRP level, fever, and larger MDSB on admission as risk factors for failure of conservative ASBO management. The risk of ASBO recurrence was not increased by surgical intervention. Although these results should be interpreted with caution, these findings might assist clinical practice. Conversion to operation increases the length of hospital stay, hospitalization costs, and intensive care unit admission.

## 1 Introduction

Adhesive small bowel obstruction (ASBO) is a major cause of emergency admission for acute abdominal conditions and accounts for approximately 20% of all surgical emergencies (1, 2). ASBO is caused by bands of scar tissue (adhesions) that form between intestinal loops or between the intestine and surrounding structures creating a mechanical blockage, presenting with abdominal pain, vomiting, abdominal distension, and failure to pass flatus or stool (3, 4). Postoperative adhesions are the leading cause of small bowel obstruction, responsible for approximately 65% of cases (5). As a nearly inescapable sequela of abdominal surgery, postoperative intra-abdominal adhesions begin to form within several hours of the operation (6). Fortunately, most adhesions remain subclinical. However, approximately 2.5% and 7.6% of patients undergoing abdominal surgery develop ASBO. The incidence varies with different operations, and pelvic procedures are associated with the highest risk of adhesions (7–9).

For fear of forming additional adhesions due to laparotomy and increased surgery associated morbidity and mortality, the surgical mantra *never let the sun rise or set on a small bowel obstruction* has not been the predominant approach. Currently, nonoperative management is the initial established standard of care for ASBO in the absence of absolute contraindications at admission (e.g., peritonitis, free intraperitoneal air, or unequivocal signs indicative of bowel ischemia) (10). Most patients with ASBO can be safely managed with conservative measures including bowel rest with nil per os, nasogastric decompression, resuscitation with intravenous fluids, and close observation. However, despite standard conservative management for ASBO, certain patients not only fail to respond but may even progress to delayed strangulation. In such patients, timely surgical intervention is imperative, as delays can lead to catastrophic consequences. Therefore, the balance between conservative management and surgical intervention is crucial.

The challenge in ASBO management is predicting which patients will fail and need earlier laparotomy before strangulation during conservative management. Current treatment decisions are often driven by the preference of the surgeon rather than following standardized, evidence-based protocols. Proactive early operation may lead to increased costs and unnecessary operative risks for certain patients, whereas postponing the procedure compromises prognosis for others (11). Although this problem has been intensively studied, the debate is still open and no consensus has been reached (2, 12, 13). While the Bologna guidelines recommend criteria for defining failure of nonoperative management, these criteria are not absolute and controversy is often present in ASBO management (14–16).

Therefore, the clinical features of patients with ASBO who fail conservative management require further investigation. Based on this premise, in this study, we aimed to identify the risk factors for conversion to laparotomy in adult patients with ASBO who initially received conservative management, and observe the outcomes following the procedure.

## 2 Materials and methods

### 2.1 Study design and eligibility

The medical records of patients admitted for ASBO between January 2017 and December 2025 at the Ningbo Municipal Hospital of Traditional Chinese Medicine (TCM), Affiliated Hospital of Zhejiang Chinese Medical University were retrospectively reviewed. The inclusion criteria were as follows: diagnosis of small bowel obstruction confirmed by abdominal computed tomography (CT), previous abdominal or pelvic surgery, age 18 years or older, complete electronic medical records, and initial conservative treatment. The exclusion criteria were small intestinal tumors; obstruction with a bezoar; incarcerated abdominal wall hernia; inflammatory bowel disease; peritonitis; intestinal perforation; and intestinal strangulation or ischemia on admission. This study was approved by the Ethics Committee of the Ningbo Municipal Hospital of Traditional Chinese Medicine (TCM), Affiliated Hospital of Zhejiang Chinese Medical University (No. 2026-015-01). This retrospective study was conducted in accordance with the principles of the Declaration of Helsinki. The requirement for informed consent was waived due to the retrospective design of the study.

### 2.2 Diagnosis of ASBO

Included patients had a history of abdominal or pelvic surgery and presented with abdominal pain, vomiting, distention, and/or constipation. All patients underwent an urgent plain abdominal CT scan to confirm the diagnosis of ASBO while excluding other etiologies.

### 2.3 Conservative management of ASBO

Once a diagnosis of ASBO was made, the patient was promptly assessed to determine whether conservative treatment was appropriate. Patients without signs of complicated obstruction were deemed eligible for conservative management based on their clinical stability. This was defined as the absence of evidence of bowel strangulation, perforation, generalized peritonitis, or other conditions requiring immediate surgical intervention at presentation.

Patients with ASBO who met the criteria for conservative management were managed with fasting, gastrointestinal decompression, and intravenous fluid and electrolyte resuscitation. Patients with fever and leukocytosis were administered antibiotics against gram-negative organisms and anaerobes. Close monitoring included abdominal pain score, temperature, abdominal examination for localized tenderness and bowel sounds, complete blood count, and volume and quality of the nasogastric tube output. The monitoring frequency was adjusted according to the clinical response in each patient. Repeat CT was performed at the discretion of the attending surgeon when clinical deterioration occurred or symptoms did not improve.

### 2.4 Definition of different treatment outcomes

Successful conservative treatment was defined as resolution of the symptoms of the patient following conservative measures within 72 h, as evidenced by reduced nasogastric output, absence of fever and leukocytosis, resolution of abdominal tenderness, and return of flatus and bowel movements. Moreover, follow-up upright abdominal radiography or repeat CT demonstrated ASBO resolution. Next, the nasogastric tube was removed and patients started oral intake. Patients who showed clinical improvement and were discharged after conservative management were classified into the conservative treatment success group (group A).

However, if the symptoms did not improve or worsened within 72 h, the decision to proceed with exploratory laparotomy was made by the attending surgeon, based on relevant physical signs and examination findings including increasing leukocytosis, persistent or worsening abdominal tenderness, or CT findings. Failure of conservative management was defined as the need for surgical intervention during the same hospitalization. Intraoperatively, the decision to perform small bowel resection depended on relevant intraoperative findings. Adhesiolysis was performed when the small intestine was dilated without necrotic changes. If the small intestine underwent ischemic necrosis, small bowel resection was performed, followed by anastomosis. Operated patients were assigned to the conservative treatment failure group (group B).

### 2.5 Discharge criteria

Criteria for discharge included stable vital signs, ability to eat and drink normally without abdominal pain or bloating, resumption of flatus or bowel movements, soft abdomen without tenderness or rebound pain, and upright abdominal radiography or CT showing no intestinal dilation or air-fluid levels, if performed.

### 2.6 Follow-up regimen

Follow-up was conducted via outpatient clinic visits at 3, 6, and 12 months post-discharge, and annually thereafter. Telephone follow-up was performed for patients unable to attend in-person visits. Recurrence was defined as re-admission for ASBO requiring conservative management or surgery.

### 2.7 Baseline clinical data collection

The following data were collected: age, sex, body mass index (BMI), number of prior episodes of ASBO, number and type of prior abdominal operations, white blood cell (WBC) count, C-reactive protein (CRP) level, blood lactate level, serum potassium level, serum sodium level, blood urea nitrogen to creatinine ratio, temperature, numeric pain rating scale, CT evidence of intra-abdominal fluid, maximum diameter of dilated small bowel (MDSB) on CT, and number of comorbidities. Data were obtained from the initial examination at hospital admission. Data regarding patient outcomes were also recorded, including the length of hospital stay, total hospitalization cost, intensive care unit (ICU) admission, and recurrence of intestinal obstruction after discharge.

### 2.8 Statistical analysis

Statistical analyses were performed using SPSS (version 23.0; SPSS Inc., Chicago, IL, USA). Continuous variables were compared between the two groups using the Student’s *t*-test, while categorical variables were assessed using the chi-square or Fisher’s exact tests. To identify factors associated with conservative ASBO treatment failure, variables of potential relevance were examined using univariable exact logistic regression. Variables retaining an association (*P* < 0.10) were included in the subsequent multivariable logistic regression analysis. Statistical significance was defined at two-tailed *P*-values of *P* < 0.05. The performance of the final binary logistic model was evaluated using receiver operating characteristic (ROC) curve analysis.

## 3 Results

### 3.1 Patient characteristics

**FIGURE 1:**
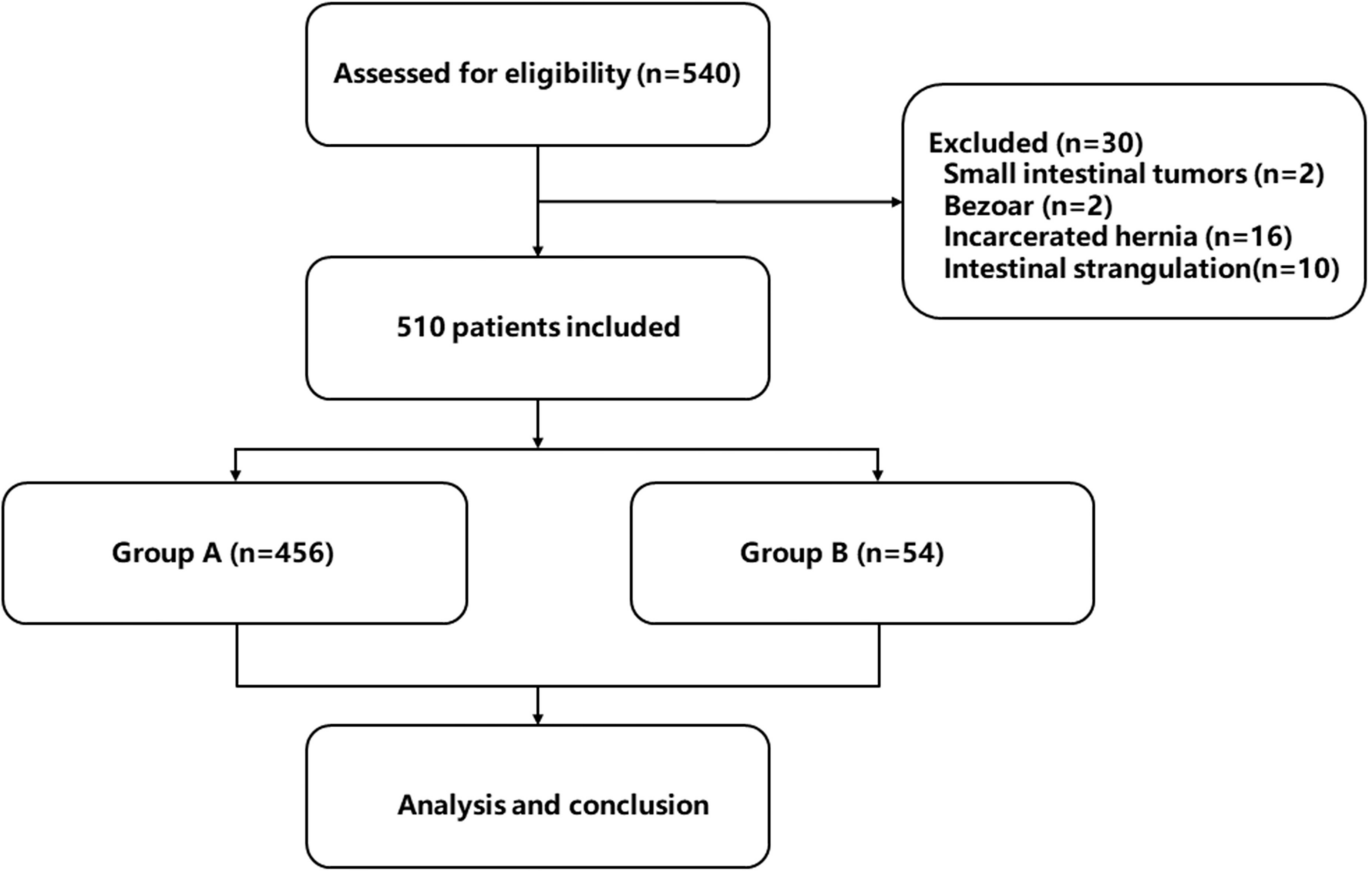
Patient enrollment flowchart.

A total of 510 patients with ASBO were included in this study, with a mean age of 66.8 ± 13.1 years. The patients were divided into groups A (*n* = 456) and B (*n* = 54). Overall, 10.5% of the patients required surgery after conservative management failure. The study algorithm and analytical process are illustrated in Figure 1.

The patient demographics and clinical characteristics were compared between the two groups for categorical and continuous variables. No significant differences were observed in sex, BMI, history of prior ASBO episodes, types of previous abdominal operations, laboratory markers (WBC count, lactate dehydrogenase level, potassium, sodium, and blood urea nitrogen to creatinine ratio), numeric pain rating scale (NPRS) scores, presence of intra-abdominal fluid on CT, or the number of comorbidities (*P* > 0.05). However, significant differences were identified in age, number of prior abdominal operations, CRP level, body temperature, and MDSB (*P* < 0.05; Table 1).

**TABLE 1:** Demography and clinical characteristics of patients between the two groups.

Variables	Group A (*n* = 456)	Group B (*n* = 54)	*P* value
Age (years)	66.2 ± 13.3	71.9 ± 10.4	0.003
Sex (*n*, %)			0.155 0.116
Male	298 (65.4%)	30 (55.6%)	
Female	158 (34.6%)	24 (44.4%)	
BMI	23.8 ± 2.5	23.3 ± 2.6	
Number of prior episodes of ASBO (*n*, %)			0.586
0	280 (61.4%)	37 (68.5%)	
1	128 (28.1%)	12 (22.2%)	
≥ 2	48 (10.5%)	5 (9.3%)	
Number of prior abdominal surgeries (*n*, %)			0.008
1	292 (64.0%)	26 (48.1%)	
2	148 (32.5%)	23 (42.6%)	
≥ 3	16 (3.5%)	5 (9.3%)	
Type of previous abdominal surgeries (*n*, %)			0.308
Upper abdomen	151 (33.1%)	15 (27.8%)	
Lower abdomen	79 (17.3%)	10 (18.5%)	
Pelvic region	166 (36.4%)	17 (31.5%)	
Combined	60 (13.2%)	12 (22.2%)	
WBC ( × 10^9^/L)	9.2 ± 2.2	9.4 ± 1.9	0.465
CRP level (mg/L)	48.4 ± 23.4	61.3 ± 17.2	< 0.001
Blood LDH level (IU/L)	179.0 ± 21.3	181.4 ± 18.9	0.427
Serum potassium level (mmol/L)	4.3 ± 0.9	4.4 ± 0.5	0.559
Serum sodium level (mmol/L)	140.0 ± 7.8	138.7 ± 5.3	0.379
BUN/Cr ratio	14.7 ± 5.2	14.3 ± 4.8	0.546
Body temperature (C°)	37.1 ± 0.5	37.8 ± 0.4	< 0.001
NPRS (*n*, %)			0.131
< 3	248 (54.4%)	25 (46.3%)	
≥ 3	208 (45.6%)	29 (53.7%)	
Intra-abdominal fluid on CT (*n*, %)	173 (37.9%)	22 (40.7%)	0.689
MDSB (cm)	3.8 ± 0.5	4.2 ± 0.6	< 0.001
Number of comorbidities (*n*, %)			0.783
0	177 (38.8%)	24 (44.4%)	
1	189 (41.4%)	22 (40.7%)	
2	61 (13.4)	5 (9.3%)	
≥ 3	29 (6.4%)	3 (5.6%)	

BMI, body mass index; WBC, white blood cell count; CRP, C-reactive protein; BUN/Cr ratio, blood urea nitrogen to creatinine ratio; NPRS, numeric pain rating scale; CT, computed tomography; MDSB, maximum diameter of dilated small bowel.

### 3.2 Risk factors for failure of conservative treatment

**FIGURE 2:**
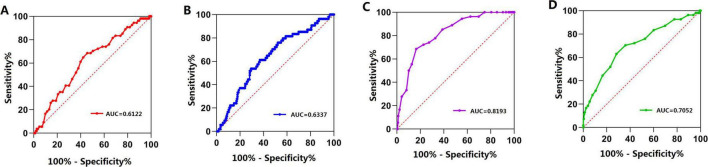
ROC curves of the logistic model. **(A)** Age: The AUC was 0.6122 (95 % CI: 0.54–0.69). At the optimal cut-off value of ≥ 68 years, the sensitivity and specificity were 68.5 % and 43.6 %, respectively. **(B)** C-reactive protein level: The AUC was 0.6337 (95 % CI: 0.56–0.71). At the optimal cut-off value of ≥ 48.4 mg/L, the sensitivity and specificity were 70.4 % and 49.6 %, respectively. **(C)** Body temperature: The AUC was 0.8193 (95 % CI: 0.77–0.87). At the optimal cut-off value of ≥ 37.7 C°, the sensitivity and specificity were 80.0 % and 37.1 %, respectively. **(D)** Maximum diameter of the dilated small bowel: The AUC was 0.7052 (95 % CI: 0.63–0.78). At the optimal cut-off value of ≥ 3.9 cm, the sensitivity and specificity were 70.4 % and 41.0 %, respectively. ROC, receiver operating characteristic; AUC, area under the curve; CI, confidence interval.

ROC curves were plotted to evaluate the predictive value of age, CRP level, temperature, and MDSB, yielding areas under the curve (AUCs) of 0.6122, 0.6337, 0.8193, and 0.7052, respectively (Figure 2). Univariable analysis was conducted on variables that demonstrated statistical significance, including age, multiple prior abdominal operations, CRP level, body temperature, and MDSB (Table 2). Variables with values of *P* < 0.10 were subjected to further multivariable logistic regression analysis. We observed that older age [odds ratio (OR) 1.035, confidence interval (CI) 1.006–1.065, *P* = 0.019], higher CRP level (OR 1.024, CI 1.008–1.041, *P* = 0.003), elevated body temperature (OR 9.812, CI 4.855–19.831, *P* < 0.001), and MDSB (OR 4.860, CI 2.342–10.085, *P* < 0.001) were independent risk factors for conservative treatment failure in patients with ASBO (Table 3).

**TABLE 2:** Univariable logistic regression analysis of risk factors for failure of conservative treatment.

Variables	B	S.E.	Wald	OR	95%CI	*P* value
Age	0.035	0.012	8.794	1.036	1.012–1.060	0.003
Multiple prior abdominal surgeries	0.550	0.211	6.763	1.733	1.145–2.622	0.009
CRP level	0.019	0.006	8.644	1.019	1.006–1.031	0.003
Body temperature	2.526	0.355	50.603	12.505	6.235–25.082	< 0.001
MDSB	1.658	0.308	28.904	5.249	2.868–9.607	< 0.001

CRP, C-reactive protein; MDSB, maximum diameter of dilated small bowel.

**TABLE 3:** Multivariable logistic regression analysis of risk factors for failure of conservative treatment.

Variables	B	S.E.	Wald	OR	95%CI	*P* value
Age	0.035	0.015	5.585	1.035	1.006–1.065	0.019
Multiple prior abdominal surgeries	0.418	0.263	2.529	1.519	0.907–2.542	0.112
CRP level	0.024	0.008	8.858	1.024	1.008–1.041	0.003
Body temperature	2.284	0.359	40.460	9.812	4.855–19.831	< 0.001
MDSB	1.581	0.372	18.024	4.860	2.342–10.085	< 0.001

MDSB, maximum diameter of dilated small bowel.

### 3.3 Perioperative findings

All patients in group B received exploratory laparotomy. The median time from admission to surgery was 2.4 days, including 35 (64.8 %) and 19 (35.2 %) patients presenting clinical deterioration and no improvement after 72 h conservative management, respectively. Bowel necrosis was confirmed in the case of 10 (18.5 %) patients. Two (3.7 %) patients were diagnosed with bowel perforation. Forty-one (75.9 %) patients were identified with bowel ischemia. Thirty-two (59.3 %) patients received bowel resection during the surgery. Postoperatively, the complications were as follows: 8 (14.8 %), 5 (9.3 %), and 1 (1.9 %) patients with surgical site infections, ileus, and anastomotic leakage, respectively.

### 3.4 Outcomes between groups

No deaths occurred in the cohort. The median follow-up duration was 28.3 months. During this period, 164 of 510 patients (32.2 %) experienced ASBO recurrence. The two groups had similar recurrence rates during follow-up (*P* > 0.05). However, patients in group B had a notably longer hospital stay (9.4 ± 2.5 vs 6.5 ± 1.5 days, *P* < 0.001), higher hospitalization cost (12,988.8 ± 1596.0 vs. 6,562.8 ± 1427.0 RMB, *P* < 0.001), and higher ICU admission rate (7.4% vs. 0, *P* < 0.001) than those in the group A (Table 4).

**TABLE 4:** Comparison of outcomes between the two groups.

Variables	Group A (*n* = 456)	Group B (*n* = 54)	*P* value
LOS (days)	6.5 ± 1.5	9.4 ± 2.5	< 0.001
Hospitalization cost (RMB)	6,562.8 ± 1427.0	12,988.8 ± 1596.0	< 0.001
ICU admission (*n*, %)	0	4 (7.4%)	< 0.001
Recurrence (*n*, %)	142 (31.1%)	22 (40.7%)	0.167

LOS, length of hospital stay; ICU, intensive care unit.

## 4 Discussion

ASBO is the most common type of small bowel obstruction encountered clinically and often results from prior abdominal operation, trauma, or inflammation (5, 17). The management of ASBO is guided by a comprehensive assessment of the patient’s clinical presentation, laboratory findings, and imaging results. Based on current evidence, approximately 70–80% of patients without signs of peritonitis or bowel ischemia can be safely managed with nonoperative treatment (3, 15). However, a subset of patients with ASBO fail to respond to conservative treatment and require emergency surgery. Some patients ultimately experience severe consequences of intestinal resection due to delayed laparotomy. The paucity of evidence concerning the determination of the optimal surgical timing before conservative management fails remains a significant gap. Therefore, investigating the clinical characteristics of patients with ASBO in whom conservative treatment will fail is important.

The reported success rate of conservative ASBO management varies considerably between developed and low-resource settings. In high-income countries, conservative management success rates typically range from 65 % to 80 %, mostly due to advanced multidetector CT with high sensitivity for strangulation, standardized water-soluble contrast protocols, availability of nasogastric tubes and total parenteral nutrition, and intensive care monitoring (18). In contrast, low-resource settings often lead to lower success rates and higher rates of bowel strangulation at presentation, mainly due to delayed hospital admission and limited access to CT (19). A study from a tertiary center in Ethiopia reported a success rate of 45.1 % for initial conservative management, with failure strongly associated with delayed presentation (3). In this cohort, 10.6% of patients with ASBO required laparotomy due to failure of conservative management. This rate was notably lower than those reported in previous studies (20, 21). This may be attributed to our thorough assessment of patients suitable for conservative treatment and the high selectivity applied to this specific group of patients. Although most patients who required operative management presented with symptoms of intestinal strangulation and intraoperative findings showed darkened intestinal coloration, some avoided bowel resection after adhesiolysis. This was achieved by wrapping the affected intestinal segments with warm saline-soaked gauze for 5–10 min, which gradually restored normal coloration and peristaltic activity. However, if operative management is delayed, prolonged ischemia in the affected bowel segment inevitably leads to intestinal necrosis. This outcome underscores the importance of close monitoring during conservative treatment and timely surgical intervention in this patient group.

This study showed that older age was a risk factor for failure of conservative management in patients with ASBO, similar to previous reports (2, 22). Patients who underwent bowel resection were older than those who underwent adhesiolysis in this cohort. In our experience, older patients present with less pronounced symptoms and signs of ASBO than younger individuals, largely due to the overall decline in physiological function with age. Intestinal necrosis and subsequent resection generally become inevitable when shock and hypotension occur. The higher prevalence of comorbidities among older patients and the prolonged operative time due to bowel resection increase the risks associated with both the operation and anesthesia. In addition, these patients are at a substantially higher risk of poor outcomes. Studies have demonstrated that a surgical delay exceeding 4 days is associated with a greater than 60% increase in mortality (23, 24). Therefore, timely surgical intervention is the cornerstone of ASBO management.

Larger MDSB was a risk factor for failure of nonoperative treatment in patients with ASBO, which aligns with the findings of Sun et al. (23). When complete ASBO occurs, the pressure within the intestinal lumen increases. This leads to intestinal dilation and compromises blood circulation. The barrier function of the intestinal mucosa is impaired in severely dilated bowel loops, resulting in bacterial translocation. Increased body temperature and elevated levels of inflammatory markers may occur. If further small bowel strangulation develops into necrosis and perforation, it can cause severe intra-abdominal infection, leading to septic shock. Our study suggests that the higher CRP level, rather than the WBC count, is associated with conservative treatment failure. This finding is inconsistent with the findings of previous studies (5, 23, 25). We believe this discrepancy is attributed to the older age of the patients in our cohort who required conversion to operative management. A decline in immune function in older patients may lead to diminished bone marrow response for the generation and release of WBCs during infection (26). Consequently, even in the presence of a severe infection, the WBC count may appear normal, creating a misleading picture.

In this study, successful conservative treatment of ASBO was associated with a shorter length of hospital stay, fewer ICU admissions, and lower hospitalization costs than surgical conversion. Follow-up after discharge indicated no significant difference in the recurrence rate of intestinal obstruction between the two patient groups. This finding appears to contradict the previous belief that laparotomy further aggravates intra-abdominal adhesions. Our findings show that the number of prior operations did not affect the success rate of conservative treatment in patients with a history of abdominal surgery. Moreover, conversion to laparotomy in this cohort did not increase the risk of ASBO recurrence. This may be related to our adoption of gentle intraoperative manipulation, meticulous hemostasis, thorough peritoneal irrigation before abdominal closure, and use of anti-adhesion materials. Encouraging early postoperative ambulation also plays a role.

This study retains several limitations due to its retrospective nature. First, this was a single-center study with a limited sample size. Second, data selection bias might exist in medical record reviews. Third, water-soluble contrast challenge was not used in this cohort. Therefore, we could not evaluate whether its use would have altered the rate or timing of surgical conversion. Future prospective studies incorporating standardized contrast protocols are warranted to complement our findings. Fourth, follow-up duration was not long enough in this cohort, late recurrences might have thus been missed in certain patients.

In summary, our study revealed that conservative management failure in adult patients with ASBO was associated with older age, higher CRP level, fever, and larger MDSB on admission. Conversion to surgical treatment led to longer hospital stays, higher hospitalization costs, and more ICU admissions without increasing the risk of ASBO recurrence.

## Data Availability

The raw data supporting the conclusions of this article will be made available by the authors, without undue reservation.
